# Anti-Viral Surfaces in the Fight against the Spread of Coronaviruses

**DOI:** 10.3390/membranes13050464

**Published:** 2023-04-27

**Authors:** Angelika Kwiatkowska, Ludomira H. Granicka

**Affiliations:** Nalecz Institute of Biocybernetics and Biomedical Engineering, Polish Academy of Sciences, Ks. Trojdena 4 St., 02-109 Warsaw, Poland; ankwiatkowska@op.pl

**Keywords:** polyelectrolytes, SARS-CoV-2, nanotechnology, antiviral

## Abstract

This review is conducted against the background of nanotechnology, which provides us with a chance to effectively combat the spread of coronaviruses, and which primarily concerns polyelectrolytes and their usability for obtaining protective function against viruses and as carriers for anti-viral agents, vaccine adjuvants, and, in particular, direct anti-viral activity. This review covers nanomembranes in the form of nano-coatings or nanoparticles built of natural or synthetic polyelectrolytes––either alone or else as nanocomposites for creating an interface with viruses. There are not a wide variety of polyelectrolytes with direct activity against SARS-CoV-2, but materials that are effective in virucidal evaluations against HIV, SARS-CoV, and MERS-CoV are taken into account as potentially active against SARS-CoV-2. Developing new approaches to materials as interfaces with viruses will continue to be relevant in the future.

## 1. Background

After the SARS-CoV-2 pandemic, few people need convincing about how much havoc the emergence of a new virus and its rapid and uncontrolled mutation can cause, and the pandemic has undoubtedly left its mark on many branches of human activity. Indeed, the pandemic has become a catalyst for introducing changes and for developing procedures that can be used in the future to offset or mitigate the damage of future pandemics. Furthermore, the appearance of the novel coronavirus in late 2019 radically changed the foci of scientific work, influencing both the focus and direction of scientific publications. It has been reported that in 2020, coronavirus-related publications grew by a factor of 20 compared to the previous two years, with more than 130,000 researchers publishing on related topics. Moreover, open-access, peer-reviewed publications related to COVID-19 accounted for 76.6% of all publications compared to 51% of all non-COVID-19 publications [1]. Based on publications on this subject, we attempt to present materials that have the potential to aid in the fight against COVID-19 and future viruses, with a particular emphasis on polyelectrolytes.

Most COVID-19-related deaths, especially in patients over the age of 65, were linked with chronic comorbidities [2,3]. In addition, it has been reported that pre-existing neurological, cardiovascular, or cerebrovascular diseases influence the progression of COVID-19 [4,5]. Infection with the virus may also impact the pre-existing comorbidities, though, disturbing the ongoing treatment. For that reason, comprehending the relationship between comorbid conditions and coronavirus disease is crucial to reducing morbidity and overall mortality among patients, especially in the elderly population. However, the correlation between risk agents and the course of the coronavirus is still not described exhaustively in the literature. Therefore, further studies and acknowledgment of the virus mechanisms of action might help develop better treatment procedures/clinical trials.

Another issue influencing the therapy is the possible interaction between the medications that treat chronic issues and the coronavirus disease. For example, Rai et al. noticed that the hydroxychloroquine and azithromycin that were applied as treatments of COVID-19 have pro-arrhythmic effects, inducing cardiovascular complications in patients [2]. Therefore, this treatment method should always be personalized and chosen carefully based on the general condition of the organism as well as the drugs already being taken. 

Pre-existing cardiovascular illness significantly enhances the coronavirus disease’s severe course, with reports showing a rate as high as by 4.8-fold [4]. Moreover, Zhou’s group study demonstrated a 10.5% death rate among individuals with cardiovascular problems [4]. In addition, factors such as the patient’s older age, systemic inflammation, immobility, severe diseases, and multiorgan dysfunctions can also raise the risk of complications [5]. The most common cardiovascular manifestations caused by COVID-19 are myocardial injury [6,7], myocarditis [8], arrhythmias [9], heart failure [10], cardiomyopathy [8], venous thromboembolic events [11,12], and acute myocardial infarction [13,14].

It is well known that the nervous system is the most under the virus attack. The loss of taste and smell and impaired consciousness were prevalent symptoms of COVID-19 among patients infected by the first virus variants. Moreover, coronavirus disease’s other neurological manifestations are agitation [15], encephalopathy [15], confusion [16], neuropathies [17], seizure [18], headache [19], dizziness [19,20], anosmia [21], ageusia [22], and ataxia [23]. Additionally, sleep-related issues are observed in patients infected by coronavirus disease [15].

In the same manner as MERS-CoV and SARS-CoV viruses, SARS-CoV-2 also demonstrates a neuroinvasive nature, entering the brain via direct and indirect pathways [24]. The first penetration route to the nervous system is through blood circulation [25]. The following access channels include retrograde axonal transport [26] and sympathetic afferent neurons of the enteric nervous system (ENS) [27]. Moreover, the virus might use the synapse-connected route through peripheral nerve terminals of the respiratory network [28]. Finally, SARS-CoV-2 infects epithelial cells of the blood-cerebrospinal fluid barrier (B-SFB) [26] and endothelial cells of the blood-brain-barrier (BBB) [29].

It was shown that the severity and fatality of coronavirus disease are correlated with pre-existing neurological and cerebrovascular comorbidities [30]. Romagnolo et al. reported that individuals with neurological issues showed a lower 30-day survival rate. Scientists draw conclusions based on cohort studies, and it is worth mentioning here that they considered different types of diseases, such as cerebrovascular or neurodegenerative disorders [31]. Furthermore, studies on patients with Alzheimer’s disease indicated a growing risk for 28-day fatality from coronavirus illness. Conversely, a similar trend was not observed for the group with Parkinson’s disease [32]. On the other hand, neurological disorders might also influence the danger of coronavirus disease morbidity. For example, the Wang group analysis showed that dementia significantly increased the risk of COVID-19 [33].

Cancer patients are also at increased risk of the severe course of COVID-19. The prognosis varies with the type of cancer; for example, hematological malignancies are associated with worse outcomes than solid tumors [2,34]. Generally, the patients undergoing cancer therapy are debilitated and burdened with the side effects of the treatment (such as immunosuppression and anemia), making their bodies less able to fight the virus. According to a COVID-19 and Cancer Consortium report based on World Health Organization (WHO) estimates, 30-day mortality in cancer patients with coronavirus disease ranges from 13–33% compared with 0.5–2% in the general population. Nonetheless, it should also be mentioned that the report’s authors concluded that some chemotherapy regimens were related to high all-cause mortality [34].

People with diabetes are another group of patients particularly exposed to a more severe course of COVID-19. Malik et al. described how diabetes Mellitus increases the chance of SARS-CoV-2 infection and affects the deterioration of the coronavirus desire course: according to the authors, the key points are weakening innate immunity, inducing an excessive pro-inflammatory cytokine reaction, and reducing ACE-2 expression [5]. Moreover, COVID-19 exacerbates sugar levels in patients suffering from diabetes mellitus. Some hypotheses assume that the direct beta-cell destruction induced by viruses might cause that process [5,35]. In addition, hypokalaemia and the enhanced resistance to insulin via fetuin-A and cytokines might worsen sugar levels [36,37]. Finally, the corticosteroids and lopinavir/ritonavir—drugs used in COVID-19 therapy––might be the root of dysglycemia [37,38]. Furthermore, it was reported that the application of angiotensin-converting enzyme inhibitors (ACEi) and angiotensin-receptor blockers (ARBs) (renoprotective and anti-hypertensive medicines) in patients with diabetes mellitus might increase the severity of coronavirus disease [4,37,39].

Coronavirus disease might also influence other organs. Thus, the associated comorbidities of COVID-19 include many more chronic diseases, such as lung [40] and kidney [41] disorders. It should be noted that the long-term consequences of the infection are still not yet known; consequently, the other undesirable effects might be revealed with time.

Due to the urgent need to develop tools for adequate anti-infective protection, the proven solutions tested in the past, and the new areas remaining only in academic disputes have all received a great deal of attention. There have been many studies whose authors, based on the available literature and experience gained in research on other viruses or bacteria, predicted the potential impact of materials on new coronavirus variants. Unfortunately, due to the lack of long-term research, some conclusions remained only in assumptions that require in-depth analysis. Only then will it be possible to assess the adequacy of potentially valuable materials. Nevertheless, the initial screening of materials cannot be overlooked. It is no different in the area of anti-virus surfaces: the first major studies on this topic were associated with earlier research on related viruses, and they became the starting point for experiments conducted on the SARS-CoV-2 virus.

Many different methods are applied to reduce the spread of COVID-19. Considering the possible infections, the most important is developing novel materials and technologies that reduce viral infectivity and spread. Personal protective equipment may be the first barrier to reducing this risk.

Thus, the development of novel materials and technologies to decrease viral availability, viability, and infectivity and to improve therapeutic outcomes can positively impact the prevention of this disease.

In this case, great hopes rest on nanotechnology and its achievements.

## 2. Coronavirus Disease Characteristics

**Figure 1:**
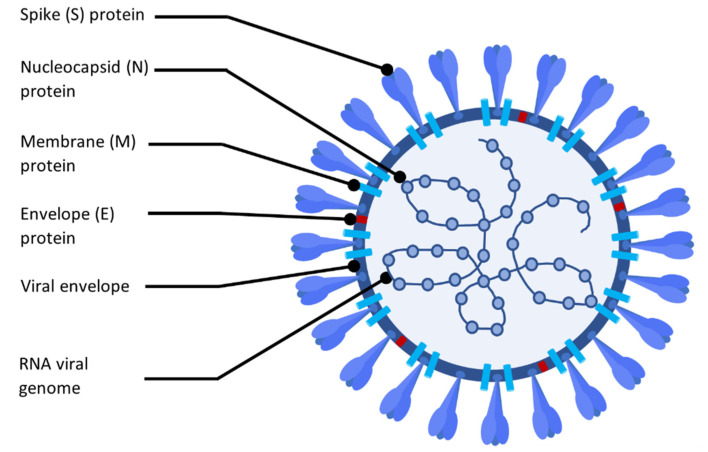
Simplified structure of the coronavirus.

Coronaviruses belong to the Coronaviridae family in the Nidovirales order [42]. This class of viruses cause respiratory discharges in animals, including humans [43]. The core of the Coronaviruses (CoVs) is built on a long positive-sense single-strand genomic RNA surrounded by a protective phosphorylated nucleocapsid (N) protein. A lipid-protein membrane further encapsulates the core. The specific viral proteins, including the envelope (E), membrane (M), and spike (S) proteins, are embedded into that membrane bilayer [44] (Figure 1).

SARS-CoV-2 is a betacoronavirus, meaning it belongs to the second genera of Orthocoronavirinae—one of the Coronaviridae subfamilies. It is worth mentioning that other viruses from that group (examples of which might be acute respiratory syndrome coronaviruses, SARS-CoV, and the Middle East respiratory syndrome coronaviruses, MERS-CoV) have triggered enzootic respiratory outbreaks previously [42,45]. The SARS-CoV-2 genome organization is shared with SARS-CoV (79%) and MERS-CoV (50%) [46]. Most SARS-CoV-2 encoded proteins are comparable to the corresponding proteins in SARS-CoV (similar length), and the amino acid identity of the four structural genes is estimated at 90%. The only exception is the S gene [47]. The SARS-CoV-2 S fusion protein is a homotrimeric glycoprotein built of two subunits—the surface, S1, and the transmembrane, S2. This protein has a full size of 1273 amino acids, and it shares high amino acid sequence similarities with pangolin coronaviruses (90.7–92.6%), bat coronaviruses in the same subgenus (76.7–77.0%), and SARS-CoVs from humans and civets (76.7–77.0%) [43]. Protein S subunits contain the heptad repeat and fusion regions as well as the receptor-binding domain [48]. The S protein plays a key role in virus entry because it can bind the ACE2 enzyme (the one converting the angiotensin) on the host cell’s surface [49].

COVID-19 is a contagious respiratory illness caused by SARS-CoV-2. It is assumed that the average incubation period of COVID-19 from exposure to symptoms is 7–14 days. As Qu and Chen reported, a few symptoms can be enumerated regarding the typical clinical characterization of COVID-19 [50]. First, although the lungs are the main target of the disease, the other organs and systems might also be afflicted. Second, it was proved that some infected individuals recover in 1 week, showing mild onset symptoms with fever. Third, it was observed that sometimes, even though obvious symptoms (such as fever, dyspnea, or coughing) do not show up in the first stages of the infection, the disease still develops (the chest imaging manifestations of such patients continue to progress), causing the exacerbated condition within the week. Dyspnea occurs 1 week after the onset in about half of the patients; in extreme cases, septic shock, coagulopathy, and acute respiratory distress syndrome (ARDS) appear. Other symptoms such as hard-to-correct metabolic acidosis and the failure of multiple organs are also present. Fourth, severe or critical patients present low to moderate fever during hospitalization or do not have a fever; such concealed cases, which are not under control, might lead to nosocomial infections. Fifth, and finally, severe cases constitute 20% of all patients with symptoms. Thus, monitoring and analyzing the course of the illness are necessary to catching the deterioration of the patient’s condition in time. It is essential to stop the development of severe failure and apply all possible treatments before the peak period in severe patients, as it was noticed that the recovery follows the survival of a 2–3-week peak period. Chest tightness and shortness of breath characterize respiratory failure, which might be measured by blood oxygen saturation [50].

## 3. Detection of Predictor Parameters for Prognosis of Patients with COVID-19

Some hematological, inflammatory, coagulation, and oxidative/antioxidant biomarkers as predictors for severity and mortality in COVID-19 were selected for examination of the peripheral blood of patients, which can predict both the efficacy of the treatment and patient prognosis. They include neutrophils and the neutrophil/lymphocyte ratio (NLR), platelet-to-white blood cell ratio (PWR), CRP, ferritin, and D-dimer values, plus the levels of malondialdehyde (MDA), nitric oxide (NO), and copper, and, finally, superoxide dismutase (SOD) activity level and total antioxidant capacity (TAC) level [51,52].

The oxidative stress biomarkers measurement principle is based on MDA’s forming lipid peroxidation by reaction with thiobarbituric acid (TBA) to form a measurable MDA-TBA compound assessed spectrophotometrically at 532 nm. The concentration of NO in serum can be assessed using the Griess reaction [53]. The nitrite NO2 and nitrate (NO_3_) (to which having a short half-life time NO is converted) measurement can be performed by spectrophotometrical assessment at 540 nm as the reflection of NO concentrations [54].

The antioxidative stress biomarkers assessment, such as serum SOD activity, is conducted by quercetin oxidation by O2, assessed spectrophotometrically at 406 nm [55].

It was observed that NLR, CRP, ferritin, and D-dimer values were elevated, and the levels of malondialdehyde (MDA), nitric oxide (NO), and copper were also elevated in the critical group compared to the mild group.

Reports indicate that platelet-to-white blood cell ratio (PWR) is a simple independent predictor of mortality in patients with severe COVID-19 pneumonia—non-survivors had a significantly lower PWR than survivors (15.8 vs. 29.0, *p* < 0.001) [52].

Moreover, the platelet-to-lymphocyte ratio (PLR) is proposed as a parameter [56,57,58,59]. However, further analysis of its correlation with patients’ conditions is needed [56,57,58,59,60]

Moreover, systemic inflammatory index (SII) derived from the parameters PLR and NLR is considered one of the predictor parameters [61].

## 4. Anti-Viral Surfaces and Coatings and Their Mechanisms of Action

The virus infectivity and the speed at which it spreads depends on factors such as population structure and age, transmission route, and new host availability (population density) [62]. The primary modes of microorganism transmissions include direct contact, airborne (aerosol), fomites (inanimate contaminated objects, e.g., medical equipment, clothing), oral (ingestion), and vector-borne (living organisms including arthropod vectors such as ticks and mosquitoes, other vermin, or rodents) paths [53,63,64]. COVID-19, as with a respiratory infection, is mainly transmitted between the population through contact paths and respiratory droplets [65,66].

High COVID-19 contagiousness and the constant mutation of the virus affect the efficiency of current strategies inhibiting the spread of the disease. Despite the tremendous worldwide effort and rigorous social distancing policies, the appearance of the new virus variants can dramatically multiply the people infected immediately and from day to day, especially when the outbreak appears in a place of large clusters of human beings with reduced immunity (nursing and convalescent homes, hospitals, etc.). For that reason, developing tools and materials to decrease virus transmission plays a crucial role in the fight against the pandemic, and is a chance to reduce the healthcare burden. Surprisingly, contrary to the topic of antibacterial surfaces, which is well described in the literature, anti-viral surfaces have not as yet been fully covered. Scientists have designed many novel materials with antimicrobial properties and have proposed new surface modifications assuring such activity, but viricidal and anti-viral functions have been studied much less extensively. Nonetheless, this situation is changing, especially after the recent coronavirus outbreak.

### 4.1. Detection of Viruses on the Surfaces

The virucidal activity on surfaces and the effect of anti-viral agents is assessed according to many international standards [62], such as the ISO 21702:2019(en) measurement of the anti-viral activity on plastics and other non-porous material [67], or the E1482 test method for the neutralization of virucidal agents in virucidal efficacy evaluation [68]. Furthermore, different techniques exist for detecting the presence of viruses and measuring their activity. Generally, they are categorized into three main groups: (a) gene or protein expression assays allowing to checking virus presence (real-time PCR and enzyme-linked immunosorbent assay, ELISA), (b) techniques based on the direct count of viral particles used to detect viruses (flow cytometry and electron microscopy), and (c) cell-based assays to assess the infectivity of viruses (focus forming assay, plaque assay, and end point dilution assay, which is also called tissue culture infective dose assay, TCID_50_) [62].

### 4.2. Physico-Chemical Surface Characteristics and Anti-Viral Properties

The mechanisms of action of viruses differ depending on the type, so the universal anti-viral surface does not exist. Thus, the materials and coatings for viral protection should be adjusted to the application and expected function. Moreover, the virus persistence is determined by environmental conditions, such as temperature and humidity [69], and so these aspects should also be considered when designing anti-viral coatings. Among the fundamental properties affecting the viruses’ persistence on surfaces, Rakowska et al. listed four: physical, chemical, or biological factors, and the material structure (e.g., porosity, absorption, hydrophobicity, and topography) [62]. Examples include biological agents in the presence of microorganisms (e.g., biofilm), envelope, and virus structure. Abiotic (physical) properties refer to temperature, relative humidity, surface type, and light exposure. In contrast, chemical factors include adsorption state, pH, solute, reactive ions, or the presence of specific substances and organic matter. Finally, it ought to be mentioned that more than once, viruses can be stabilized by the organic matter (mucus droplets, saliva, etc.) in their vicinity.

As previously mentioned, each virus type has a different interaction mechanism with the surface. Therefore, the specific physical properties of surfaces—porosity, absorption, and hydrophobicity [62], which play a crucial role in viruses’ survival––should be analyzed to assess the material anti-viral activity. For example, the studies demonstrated that SARS-CoV-1 and SARS-CoV-2 showed better persistence on less absorbent or hydrophobic materials (e.g., surgical masks) than on hydrophilic ones (i.e., natural fibers, such as cotton) [70]. This thesis confirmed the report of Edward’s group, which concluded that absorbent materials such as leather and polyurethane foam lowered viral recovery [71]. Although the mechanism behind the good virucidal properties of absorbent materials is not well known and requires further investigation, Owen’s group hypothesized that the enhanced moisture absorption of hydrophilic materials enhancing viral desiccation might play a crucial role here [70].

Coronaviruses generally exhibit more remarkable persistence on impermeable surfaces (stainless steel, plastic) than on porous ones (cloth, paper). Chatterjee et al. explained the correlation between the survival time of a coronavirus on non-porous surfaces and the drying time of a respiratory droplet along with the residual film that is left on those surfaces, analyzing the mechanism of bulk droplet evaporation. In the case of non-porous material, the diffusion-limited evaporation of the thin liquid film exceeds mass loss from the respiratory droplet, whereas for porous surfaces, the dominant process is the much faster capillary imbibition. According to scientists, a thin liquid film remaining on the coatings after the bulk droplet vanishes plays the role of a medium supporting the virus’s survival. It should be noted that film evaporation is slower on impermeable surfaces than on porous ones. The evaporation rate depends on disjoining pressure, which is enhanced for porous materials of the increased effective wetted area caused by voids. Moreover, the capillary action between the contact line and material fibers drives the droplet spreading on porous surfaces. To summarize, non-porous materials characterized by a high evaporation rate are more susceptible to the survival of the virus because they support rapid drying, leading to viral desiccation and, finally, inactivation [70,72].

### 4.3. Anti-Viral Materials

Knowing how the virus attaches and enters host cells allows for designing surfaces that interfere with these processes and the resultant reception of materials with better anti-viral characteristics. The virus is assumed to link to the receptor on the host cell’s surface via the viral spike protein. The pH fusion and replication then follow [62]. Certain substances can interfere with this process, preventing the virus from invading a host cell and multiplying. Another way to affect virus viability is to imbalance the elements crucial for viral proteins and processes (e.g., reverse transcription, catalysis, nucleic acid chaperoning, and folding/unfolding) [73].

**Figure 2:**
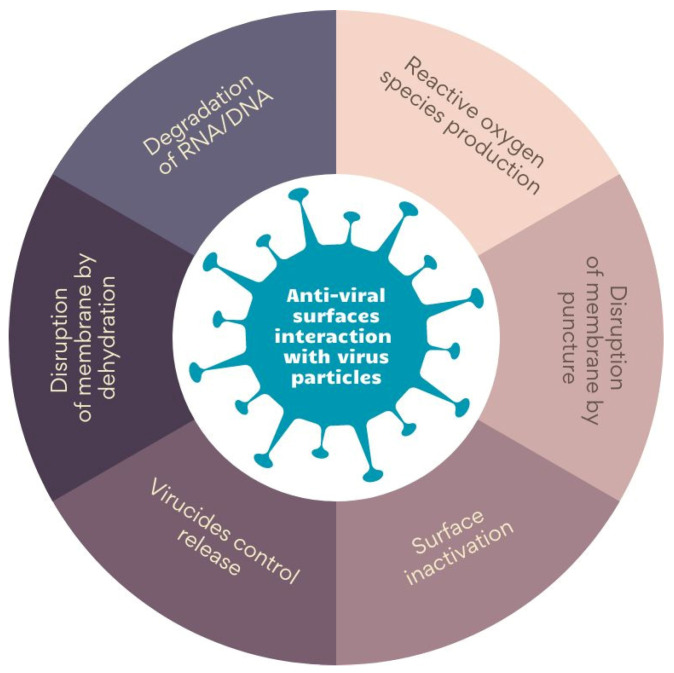
The mechanism of anti-viral surface interaction with virus particles. Prepared on the bases of ‘[a]nti-viral surfaces and coatings and their mechanisms of action’ by Rakowska et al. [62].

The literature describes various mechanisms of anti-viral surface interaction with virus particles. The main ones are collected in Figure 2. Some materials, such as graphene and its derivatives, disrupt membranes through the puncture [74], while others disarrange virus membranes by dehydration. On the other hand, photosensitizing surfaces induces the generation of reactive oxygen species (ROS), thereby damaging virus proteins [75,76]. Biopolymers, in turn, inactivate viruses by binding to the capsid proteins or membrane [77,78]. Finally, ionic surfaces of polyethyleneimine and metals cause the degradation of RNA/DNA chains. An interesting approach is using materials that allow for the controlled release of anti-viral agents—an example might be antimicrobial peptides encapsulated in hydrogels (AMPs) [78,79,80].

Although many studies in recent years have been performed on materials that are practical in the fight against SARS-CoV-2, there are still many unknowns that only long-term investigation can explain. Nonetheless, some conclusions might be drawn from the experiments on other coronaviruses. A similar approach was applied to accelerate works on the design of virucidal surfaces during the peak of the global pandemic. The attention of scientists was turned to the material traditionally considered anti-viral, such as the metal ions and graphene derivatives mentioned above, or polymers and peptides. Starting from the theoretical discussion, through in vitro studies, and ending with the clinical tests, the materials that proved effective in a virucidal evaluation against HIV, SARS-CoV, and MERS-CoV were taken into account as potentially active against SARS-CoV-2, too.

Among the considered anti-viral materials such as metals, graphene, polymers, and peptides, antimicrobial peptides (AMPs) are the less-known category of substances exhibiting anti-viral properties. However, these heterogeneous peptides provide activity against many viruses, such as influenza A viruses [81] or respiratory syncytial virus (RSV) [82]. Kurpe et al. presented a comprehensive review of the mechanism of the action of AMPs on virus particles, and, moreover, the scientists examined the potential application of peptides in the battle against SARS-CoV-2 infection [83]. Another group of anti-viral materials that have gained great popularity recently is graphene together with its derivatives (such as graphene oxide, GO, or reduced graphene oxide, rGO). The sharp edges of their surface and the characteristic structure of the flakes, with their high surface area to volume ratio, probably cause a virus membrane disruption by a puncture, and, as a result, graphene derivatives show viricidal activity [62]. Graphene-based materials were employed in biosensor construction [84,85] and used in the design of personal protective equipment (PPEs) [85], and were even incorporated into the structure of face masks [86,87].

The most famous group is metal ions, though, especially Cu ions, which can interact with viral components by inactivating their metalloproteins. For example, it was observed that cooper and cooper alloy surfaces inactivate the coronavirus, causing irreversible and nonspecific RNA fragmentation [70]. Some authors reported the anti-viral activity of metal catalysts Ag/Al_2_O_3_ and Cu/Al_2_O_3_. It was observed that after exposure to the catalyst surfaces for 5 min to 20 min, the infectivity of SARS-CoV in Vero cells and baculovirus in Sf9 cells significantly declined [88]. The anti-viral properties of zeolite (sodium aluminosilicate) powders containing Ag and Cu ions incorporated into the plastic were reported. In addition, the authors observed a significant reduction in feline infectious peritonitis virus (FIPV) and coronavirus 229 E after 24 h of exposure to the samples [89].

**Figure 3:**
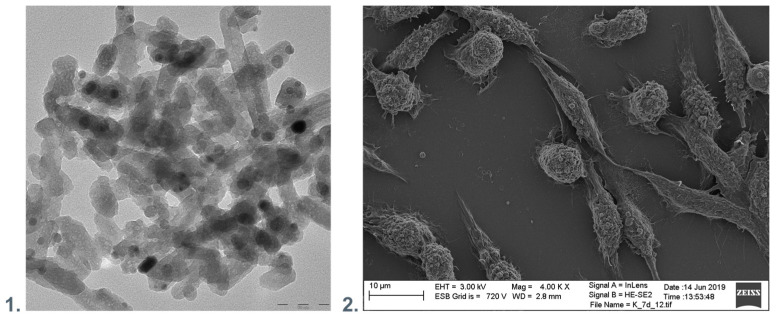
(**1**). TEM visualization of the polyethyleneimine with hydroxyapatite and silver nanoparticles incorporated. (**2**). SEM visualization of nanocomposite polyethyleneimine with hydroxyapatite and silver nanoparticles incorporated with immobilized fibroblastic cells.

Hence, nanocomposites of NPs and polymers may play an essential role in the fight against the viruses. Figure 3 presents the exemplary surface of nanocomposite polyethyleneimine with hydroxyapatite and silver nanoparticles incorporated and the material elements. Antimicrobial surfaces should preferably maintain the functions of eukaryotic cells.

It can be noted that viruses do not show metabolic activity outside a host cell. Thus, the virus inactivation does not require a metabolic process. The purpose of removing or destroying viruses to such an extent that successful reproduction in a susceptible cell is prevented can be accomplished by the immobilization of viruses on a surface, the blockage or destruction of host–cell receptors on the virus, or the inactivation of the nucleic acid within the viral capsid [90].

**Figure 4:**
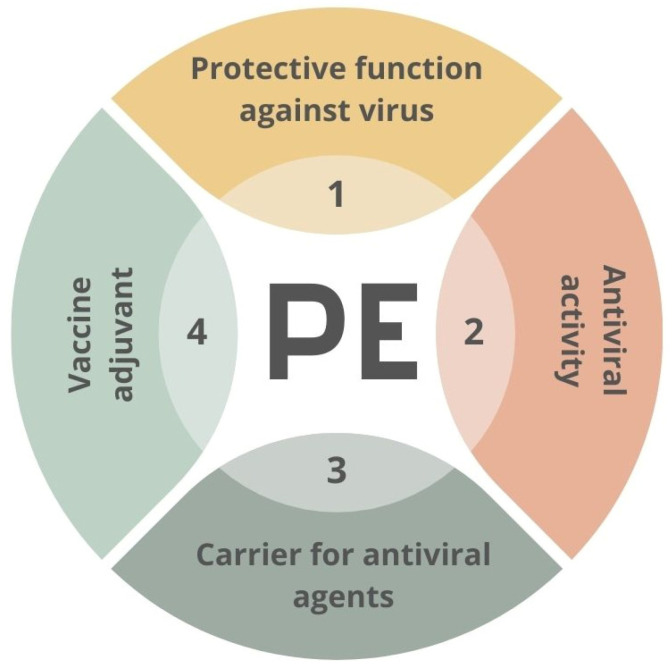
Scheme of potential usage of creating of material–virus interface. PE—polyelectrolyte.

The materials used in the fight against SARS-CoV-2 may include polymers applied in various ways [91,92]. Polymers can act as anti-viral factors interacting with the surface of the virus. However, they can also be stabilizers or carriers for other active agents’ transportation. An example of the second approach might be the polyethyleneimine-based coatings employed as a matrix for AgNPs [93] or oseltamivir-phosphate-loaded poly(lactic-co-glycolic acid) nanoparticles [94]. The potential functions of the PE–virus interface are presented in Figure 4.

Polyelectrolyte layered systems can have an essential role in anti-viral function. Experimental results indicate that the balance between ionic and hydrophobic interactions plays a leading role not only in the construction of self-assembled systems but also in the functional properties of the bioactive interface [95]. Therefore, building different multilayers based on hydrogen bonding can be introduced as a mild process, allowing the maintenance of the function of cells that come into contact with the layer [96,97,98,99], and, on the other hand, adsorbing viruses [92]. The hydrogen-bonded PE multilayer involves hydrogen interactions of the polymers using non-ionic layer-by-layer complexation. The exemplary PE pairs, which include hydrogen bondings applied as a non-toxic modification of the polymer surface–cell interactions, are the polyacrylamide (PAAm) assembled with weak polyelectrolytes poly(acrylic acid) (PAA) or polyaspartic acid (PASA), or else the biocompatible, non-ionic polymers such as poly(N-vinylpyrrolidone) (PVPON) and tannic acid (TA)—(TA/PVPON) shells bonded by hydroxyl groups of TA and carbonyl groups of PVPON explored for cell surface engineering.

There is great potential in the weak reversibility of PE systems. The multilayers assembled of weak PE have been explored, especially for drug delivery systems applications, whereas they can also be used for anti-viral functions. Some pairs of weak polyelectrolytes exist [100] and are widely employed for constructing stable multilayers in a specific pH range. Those stable over a wide pH range (2.5–11.5) are poly(methacrylic acid) (PMA) and poly(allylamine) (PAH) [101], and those that create stable multilayers in the acidic pH range are PAH/PAA [102], poly(acrylic acid) (PAA)/poly(vinyl alcohol) (PVA) [103], chondroitin sulfate/polylysine [104], poly(ethylenoxide) (PEO) and poly(N-vinylpyrrolidone) (PVPON) [105], PAA and PMA [106]. The knowledge about the pKa values of PE building the shell is crucial in constructing the shells. The mechanism of membrane pores opening/closing results from an electrostatic repulsion that causes the polymer gel to expand when its chains are charged, while when the functional groups lose their charge, the repulsion fades, resulting in the collapse of the material. It can thus be noted that such polypeptides as poly-L-lysine (PLL) and poly-L-glutamic acid (PGA) are valuable models for studying protein conformational changes, since they can adopt multiple conformations, such as α-helix, β-sheet, or random coil structures, in response to changes in the pH and temperature conditions [107].

**Figure 5:**
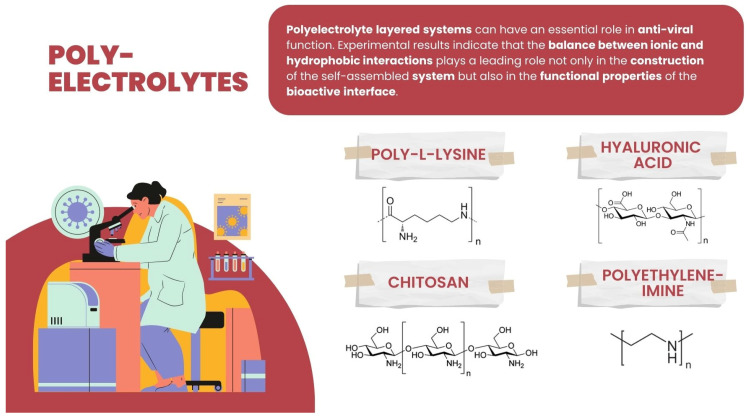
Chemical structure of hyaluronic acid, chitosan, polyethyleneimine, and poly-L-lysine.

Among PE explored and/or applied in various ways for interaction or for creating an interface with the viruses, some basic ones can be enumerated: polycationic such as synthetic polyethyleneimine (PEI), polysaccharides such as poly-L-lysine (PLL) and chitosan, and the polyanionic-like polysaccharide hyaluronic acid (HA) (Figure 5).

*Polylysine:* Polycationic compounds are considered to be essential in interacting with viruses. One of the possible strategies for their application is to inhibit the replication of viruses. Polylysines were primarily assessed for anti-virus usability [108,109,110,111]. Since the 1970s, researchers have analyzed PLL influence on different viruses, like infectious ribonucleic acids (IRNA) of Venezuelan equine encephalitis and Eastern equine encephalitis viruses, observing that these viruses formed noninfectious complexes with poly-L-lysine [108]. Thus, the polylysine function can be assigned by preventing or limiting viruses’ entry into the cells [110]. Some research was performed on poly-L-lysine’s influence on the replication of viruses, such as human immunodeficiency virus type 1 and type 2 (HIV-1 and HIV-2), respiratory syncytial virus (RSV), Influenza A virus, Sindbis virus, Semliki Forest virus, vesicular stomatitis virus (VSV), herpes simplex virus type 1 (HSV-1), and human cytomegalovirus (CMV). It was observed that poly-L-lysines of different molecular weights of 4000–26,000 Da deposited on the surface of MT-4 cells of the human T-cell line proved inhibitory to the replication of these viruses, which was explained by an inhibitory effect on the virus binding to the host cells [109]. Furthermore, recently hyperbranched polylysine nanoparticles were reported to effectively inhibit SARS-CoV-2 replication [112].

*Polyethyleneimine:* (PEI) represents synthetic polycations used as nucleic acid transfection reagents in vitro and DNA vaccine delivery vehicles in vivo. In addition, polyethyleneimines and their derivatives may be usable molecules for the development of anti-viral platforms. Some authors have studied the inhibitory effects of a linear 25-kDa polyethylenimine on infections with human papillomaviruses and human cytomegaloviruses [42]. It has been observed that the presence of the shell of the polyethylenimine on eukaryotic cells blocked the primary attachment of both viruses to the cells, significantly reducing infection. Moreover, the polyethylenimine concentrations required to inhibit human papillomavirus and cytomegalovirus did not cause cytotoxic effects [113]. Other researchers have investigated the ability of polyethylenimine, with different forms (linear or branched), to inhibit the replication of the Porcine reproductive and respiratory syndrome virus (PRRSV). It has been observed that the linear 40 kDa PEI, or the 25 kDa linear PEI, inhibited heterogeneous PRRSV-2 isolates’ influence in MARC-145 cells and primary porcine pulmonary alveolar macrophages without exerting a cytotoxic effect on these cells [114]. The direct anti-viral activity was reported by some other authors who observed that painting a glass slide with branched or linear N,N-dodecyl methyl-polyethylenimines, and certain other hydrophobic PEI derivatives, enables it to kill the influenza virus with essentially 100% efficiency (at least a 4-log reduction in the viral titer) within minutes [115].

*Hyaluronic acid* is a linear non-branched anionic polysaccharide from a group of proteoglycans, built of disaccharide units of D-glucuronic acid and N-acetyl-D-glucosamine bound alternate with glycoside bindings (in position beta-1-3 and beta-1-4). The biological properties of HA depend heavily on its molecular weight, e.g., HA with low molecular weights (60–200 kDa) exhibits immunostimulatory and angiogenic activities. On the other hand, HA with a medium molecular weight of 200–500 kDa is involved in wound repair and regeneration, and high molecular weight hyaluronic acid (>500 kDa) with hyaluronan chains can reach 2 × 10^4^ kDa and exhibits both anti-angiogenic and immunosuppressive activity. It can also impede differentiation, possibly by suppressing the cell–cell interactions or the ligand access to cell surface receptors [116,117]. Therefore, HA can be applied as the material for vaccine carriers to ensure the immunization of inactivated viruses, including SARS-Cov-2 [118]. Pre-treatment with high molecular weight hyaluronic acid allows the VERO cells infected with Coxsackievirus B5 (COXB5), mumps virus (MV), and herpes simplex virus (HSV-1) to exhibit anti-viral activity against them. On the other hand, assessing the infected MARC145 cells, no activity against the porcine reproductive and respiratory syndrome virus (PRRSV) was observed [119].

HA can be applied as the material for vaccine carriers, ensuring the immunoisolation of inactivated viruses, including SARS-CoV-2 [118].

*Chitosan* is a cationic linear polysaccharide [120] composed of randomly distributed β-(1→4)-linked D-glucosamine and N-acetyl-D-glucosamine. The chitosan products exhibit a molecular weight mainly of 3800–200,000 Daltons. Chitosan is obtained by the chemical modification of animal or fungal source materials. The variability in the physicochemical properties of chitosan due to variable sources of it as well as the preparation process can cause differential biological and anti-viral performance.

Chitosan has been reported to have a significant activity of viral inactivation [121,122]. Moreover, chitosan and its derivatives have been shown to exhibit direct anti-viral activity, to be helpful vaccine adjuvants, and to have potential anti-SARS-CoV-2 activity [123,124]. For example, chitosan nanoparticles were used for intranasal delivery of plasmid DNA encoding nucleocapsid SARS-CoV-2 for stimulation. This stimulates the secretion of the SARS-CoV-2 spike protein, which can compete with the live coronavirus for binding to human ACE2 receptors in a rodent model [124].

Moreover, considering that the virus carries a positive zeta potential, there are ideas of potentially applying the positively charged chitosan nanofibers incorporated into fabrics to produce protective clothes to decrease the viral load on the fabrics [125].

Some other polymers such as poly(lactide-co-glycolide) (PLGA) were reported as nanoparticle shells in the delivery of anti-viral factors against the HIV-1 virus [126] and PLGA-PEG-PLGA carriers against the cytomegalovirus [127]. Moreover, the PLGA nanoparticle carriers have shown promise for the targeted delivery of antiretroviral drugs to the brain [128]. Furthermore, a PLGA core coated with membrane from either human lung epithelial type II cells or human macrophages was observed to exert a neutralizing activity against SARS-CoV-2, preventing viral infection of Vero cells [129].

Surface engineering plays an essential role in modifying multilayer shell systems for modification of biological processes, such as influencing their interaction with cells and/or tissues [98,130,131,132]; regulating the essential functions, e.g., biological material adhesiveness [133,134]; cytophobicity; and inhibition of molecular recognition between complementary molecules. The approach to adsorb viruses can be the construction of a multi-shell mimicking the natural configuration of biological material, e.g., phospholipids and glycolipids of the cell membrane. An example of such a natural construction of multi-shell material is a mixture of phospholipids and cholesterol {lecithin,1,2-distearoyl-sn-glycero-3-phosphoethanolamine-N-[methoxy(poly(ethylene glycol)-2000)] (DSPE-PEG-2000), 1,2-distearoyl-sn-glycero-3-phosphoethanolamine-N-[maleimide(polyethylene glycol)-3400] (DSPE-PEG-3400-Mal), and cholesterol in a 60:10:2:15 mass ratio} forming lipid film applied as a coating shell [135]. The amphiphilic zwitterionic phosphorylcholine functionalized chitosan oligosaccharide with hydrophobic stearic acid copolymer can mimic the hydrophilic head groups of amphipathic lipids [136]. Multilayer shell capsules’ walls can be modified towards desired properties by functionalization with specific moieties. Some particles immobilized within the nano-coating may can serve as viral adsorbing surfaces. Metallic nanoparticles can be applied for this purpose [111,137]. An example is poly(ethylenimine), which can be utilized directly [138] or functionalized with Cu or Ag particles for better performance [139]. It is worth mentioning that these nanoparticles can effectively kill viruses in microfiltration membranes applied for drinking water without a significant change in the transport properties [140].

Moreover, silver NPs involved in polymers may be applied to obtain an anti-viral function. For example, the polylactide (PLA) films with incorporated silver nanoparticles were reported to influence feline calicivirus titers dependent on silver concentration [141]. The addition of silver nanoparticles to chitosan results in a composite material with anti-viral activity against the H1N1 Influenza A virus. The dose-dependent anti-viral potential was shown with complete eradication of the virus at a concentration of 300 silver nanoparticles per μg of chitosan [122].

Reports indicate that both positively and negatively charged polyelectrolytes promote interaction with viruses. In some of them, the authors postulated that the virucidal mechanism of action destroys the virus lipid envelope through “tentacle” fragments of the polyanionic chains. The outer lipid membrane of enveloped viruses can strongly adsorb polycations involving polycationic chains that, after being absorbed, can promote envelope disorganization and viral deactivation.

Isoelectric Point (pI) and, among others, the Molecular Weight (MW) of the SARS-CoV proteins can be predicted by ProtParam, including the corresponding sequence identifier. For SARS-CoV-2, the value of the isoelectric point of the spike glycoprotein is pI = 6.24 [142], implying that the stalk part is negatively charged due to a lower than physiological pH value (7.2), allowing for the electrostatic interaction of positively charged groups of anti-viral materials.

**Figure 6:**
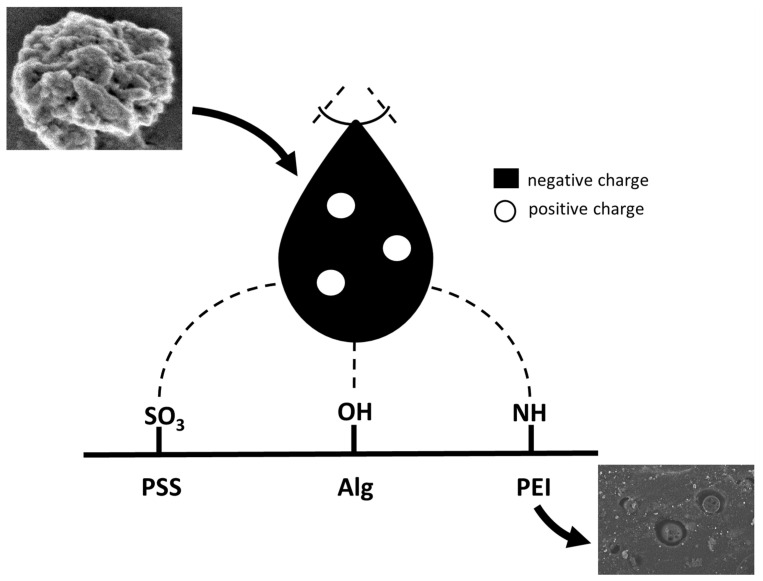
Model of electrostatic interaction of exemplary polyelectrolytes with a spike of Sars-Cov protein. SEM pictures: higher- SARS-CoV-2 protein; lower- deposited on the polysulfone support polyethyleneimine layer with hydroxyapatite and silver nanoparticles incorporated.

Simultaneously, the observed favourable mutations in SARS-CoV-2 increase the tendency to form positively charged residues in the spike protein [143]. Since a positively charged region appears, surfaces containing negatively charged functional groups can adsorb Cov. (Figure 6).

PEI and its copolymers are an example of positively charged polyelectrolytes. For example, it has been observed that polycationic glass slide coated with N,N-dodecyl-methyl-polyethyleneimine exhibited virucidal effects against Influenza A virus after 5 min of incubation [115]. On the other hand, examining the negatively charged PE, some authors have found that negatively charged dendrimers displayed an anti-viral effect against HIV-1 viruses [144].

Moreover, some research was performed on the Tobacco Mosaic virus’s anti-viral effect of negatively charged carboxylate-based polymers, such as, among others, poly(acrylic acid), poly(methacrylic acid), poly(vinyl acid) copolymers, and poly(styrene acid) copolymers [145]. It was reported that the anti-viral effect was observed in vivo in the rodent model. It can be noted here that other authors observed in vivo that the negatively charged polymers were activated only when complexed with organic cations (arginine and poly L-ornithine) [146]. In addition, poly(4-styrene sulphonic acid) (PSS) was reported to exhibit anti-viral effects on HIV-1 and HIV-2 in MT-4 cells [147]. Recently, AuNPs with PSS coating shells were reported as anti-viral agents exhibiting activity against viruses, including HIV-1, ZIKV, HSV-1, RSV, seasonal coronaviruses, and SARS-CoV-2 variants [148].

Moreover, both anionic and cationic dendrimers exhibit anti-viral activity. An example can be anionic dendrimer—astodrimer sodium, which inhibits the replication of SARS-CoV-2 in Vero E6 cells when added to cells 1 h prior to or 1 h post-infection [149]. Additionally, polyanionic carbosilane dendrimers are reported to exhibit anti-viral activity against HIV infection [150]. On the other hand, polycationic dendrimers with N-alkylated units also showed the inhibition of HIV-1 in MT-4 cells [151].

The viruses influenced by basic polyelectrolytes and/or their nanocomposites are presented in Table 1.

Polyelectrolyte materials with direct anti-viral activity against SARS-COV-2 are listed in Table 2.

### 4.4. Other Applications of PE for Viral/Bacterial Binding and Potential Usage

Some systems designed for purposes other than antimicrobial ones can find application in anti-viral systems. Such systems can involve nanoparticular systems, ranging in size from a few nanometers to several hundred nanometers, and can interact with biomolecules located on biologically active material [160]. Moreover, the involvement of PE systems can represent an interface that provides a platform for the systematic modification of biological processes by incorporation or assembly of biological, organic, and inorganic materials.

Some authors reported oxygen/water vapor-plasma-treated polished aluminum substrates coated with poly(acrylic acid) (PAA) using the deeping-coating technique and subsequently heating. It was observed that the covalently attached PAA macromolecules exhibited antibacterial characteristics, resulting in a 98% decrease in the 5-strain mixture of the Listeria monocytogenes population during 24 h of incubation, with the aluminum substrates coated with PAA. Moreover, during the incubation in the presence of this material, 82% to 96% of bacterial numbers were reduced for the mix of three different bacteria: *Pseudomonas aeruginosa*, *Staphylococcus epidermidis*, and *Escherichia coli* [161]. These systems can be approaches for anti-viral functioning/usage.

Some other authors have developed an idea based on the assumption that randomly arranged supermolecular species incorporated in a network medium can ultimately create ordered structures at the surface by regulating dynamic and equilibrium driving forces. For example, the authors observed that the competitive charge binding between M13 viruses and two oppositely charged weak polyelectrolytes lead to interdiffusion and the virtual ‘floating’ of viruses to the surface, which may allow the creation of the platform for the incorporation of this biological material [162].

## 5. Nanotechnology’s Role in Fighting the Pandemic

Undoubtedly, nanotechnology plays a significant role in battling the global pandemic; its achievements include pharmacology, theranostics, and treatment, and allows for the construction of new tools and materials to prevent the virus from spreading.

Nanomaterials are applied in coronavirus infection diagnostics in ultrasensitive biosensors and fast tests. Optical and electrochemical nanobiosensors based on affinity mechanisms are involved in SARS-CoV-2 detection. Such tools provide rapid and accurate results and lower the detection limit. In addition, affinity-based biosensors demonstrate high specificity in various bioreceptors, such as ssDNA, antibodies, or aptamers, improving their sensitivity [2]. Among the most popular materials applied in the COVID-19 biosensors, graphene [163,164,165], nanowires [166], gold nanoparticles [167,168,169], and gold nanoislands [170] can be listed. Another promising diagnostic tool for SARS-CoV-2 is the chiroimmunosensor employed for other coronavirus detections from blood samples [171,172].

The nano-sized particles are employed in developing potential therapeutics and vaccines for COVID-19. Their unique properties, such as enhanced solubility and a good target reachability, create an opportunity to improve the drug’s delivery and optimize the release time of medicaments. For example, Jermy et al. developed the nanosystem—PEGylated green halloysite/spinel ferrite nanocomposites—for pH-sensitive drug delivery. Their work focuses on dexamethasone, given that this active agent is applied in lung infection treatment of coronavirus origin. The reported results show that the carriers’ application decreases the toxic effect of Dex and enhances cell viability significantly [173].

On the other hand, Anand’s group have presented the exciting hypothesis of improving COVID-19 treatment with immunotherapeutics. They suggest using the plasma-derived exosomes from COVID-19 convalescents (convalescent plasma exosomes—CP^Exo^) as carriers for anti-viral drugs, such as Lopinavir-ritonavir and Darunavir Prulifloxacin, and, according to these scientists, it could stop the cytokine storm induced by the immune system [174].

Furthermore, nanotechnology also supports producing protective clothing and equipment to prevent the virus from spreading and fulfils a vital role in manufacturing modern disinfectant agents. Personal protective equipment (PPEs) plays the primary role in the defence against infection. Protective suits, boot covers, gloves, facemasks, goggles, visors, etc. are all used to shield people from contracting the virus. Nanomaterials can be easily incorporated into the structure of classic fabrics without a significant change in their texture, simultaneously ensuring a great improvement of other properties such as hydrophobicity or virucidal activity [175]. An excellent example is the design of face masks. Traditionally they are made of woven or non-woven materials and work on the principle of filters. The disadvantage of such an approach is that the virus particles are entrapped in the mask surface [176]. Consequently, they can be released if the mask is worn incorrectly. In modern strategies, self-cleaning masks are employed, which rely on nanosize composites [15]. For example, nanoparticles of anti-viral properties, such as copper oxide or silver nanoparticles, are incorporated into the fabrics or the fibrous membranes of the mask [177,178], and the new idea is to apply materials (graphene, metallic nanowires, fluorinated polymers) that make the surface superhydrophobic, thereby preventing virus particles from settling on the mask [179].

There is a discrepancy with the earlier studies that showed that SARS-CoV-1 and SARS-CoV-2 exhibited better persistence on less absorbent or hydrophobic materials than on hydrophilic ones. No universal model of adhesion has been developed so far. However, it can be assumed that in the interaction between the virus and the material surface, in addition to factors related to hydrophobicity/hydrophilicity, electrostatic and/or hydrogen interactions are involved. For example, the size and polarity of the charges carried by viral particles, combined with the electrostatic properties of a given surface, can regulate viral adhesion. Moreover, the electrostatic interactions can vary with pH, the concentration of the surface charge of the virus, and the isoelectric properties of surface proteins [180].

Unfortunately, not all nanomaterials can be used in PPEs with a large surface area, as their uncontrolled release into the environment, especially in high doses, has not yet been thoroughly examined [181]. Hence, there is a risk of potential contamination (metallic nanoparticles) [182]. For that reason, searching for novel anti-viral materials and composites is still a topic of interest for many scientists. Three-dimensional (3D) printing is also used to produce personal protection and medical equipment with anti-viral and antimicrobial properties. The undeniable advantage of this technology is the possibility of using various materials such as ceramics, polymers, and metals. The exciting solution might be to combine 3D printing possibilities with nanomaterial advantages [2].

Finally, nanomaterials are employed to improve the anti-viral properties of large surfaces and coatings in public settings; for example, they can be applied on walls, cabinets, or doorframes when added to the paint [183,184,185]. Using surfaces with an admixture of nanomaterials can be especially beneficial in the public transport system or healthcare facilities, where the exposition of microorganisms is relatively high, and the risk of virus transmission via fomites increases significantly. On the other hand, nanotechnology can also help to receive the hard surfaces (touch screens) of the enhanced anti-viral activity or to design the protective coatings of such a function covering them [186,187,188]. For example, Das et al. proposed a copper-graphene nanocomposite-based highly transparent coating system of anti-viral properties preserved in the solid form, which could be implemented on hard surfaces [189].

**Figure 7:**
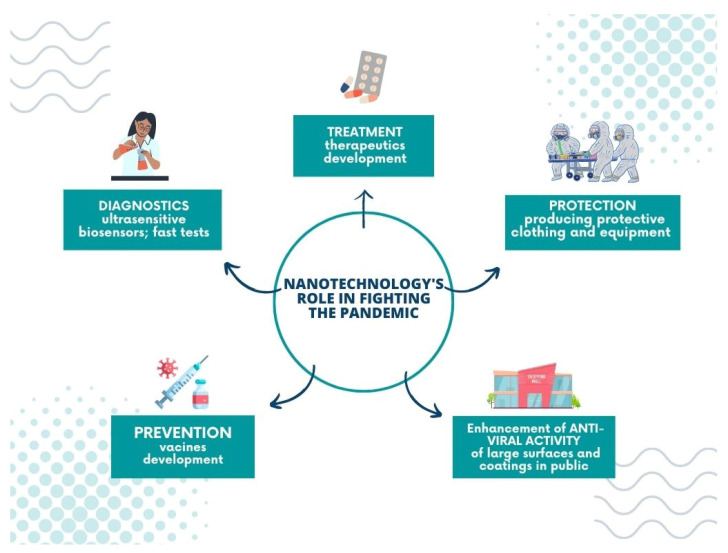
Nanotechnology’s role in fighting the pandemic.

Moreover, some authors have also studied the anti-viral properties of similar surfaces. For example, Ma and coworkers reported that ultrathin-film nanofibrous composite with cellulose nanofibers could filter virus adsorption [190]. The nano-coatings of this type may serve as an outer layer of materials for use in public areas or be applied for protective clothing production. Herein, we present the nano-coatings of anti-viral activity for application in personal protective and medical equipment (Figure 7).

## 6. Outlook

This review has aimed to provide an overview of polyelectrolyte nanomembranes and the incorporated additives applied in systems with anti-viral or potential anti-viral functions as an aspect of nanotechnology in fighting against the spread of COVID-19. In addition, recent advances in developing such systems have been mentioned, including polyelectrolyte materials applied for shell forming. The work has covered aspects such as coronavirus disease characteristics, anti-viral surfaces, coatings, and their mechanisms of action. Moreover, nanotechnology’s role in fighting the pandemic has been discussed and demonstrated.

The perfect material for a fight against viral infection does not exist. In order to select the material with the most favorable properties, it is necessary to analyze the impact of the properties on the virus, such as morphology or the presence of functional groups. In addition, knowledge of the structure of the virus protein, especially protein S subunits, is helpful when assessing how the virus attaches and enters the host’s cells, allowing for the design of surfaces that interfere with these processes and that consequently receive materials with more efficient anti-viral characteristics.

There is potential for materials that can immobilize viruses from within, which can prevent the spread of viral infections. Indeed, due to the fact that virus inactivation does not require a metabolic process, virus immobilization on a surface might be an efficient way of blocking the spread of the virus in the case of protective clothes.

Furthermore, biomaterials and the development of biotechnological systems can potentially limit viral viability and infectivity. Recent evidence suggests that materials applied in these systems are essential in this process: polyelectrolytes and metals causing degradation of RNA/DNA chains, graphene disrupting membranes through the puncture, peptides and other polymers with (among others) photosensitizing functionality that can damage the virus protein by the generation of reactive oxygen species.

Even if SARS-CoV-2 evolves into an endemic form, developing new solutions for anti-viral materials and differing vaccine adjuvants will be necessary.

## Figures and Tables

**Table 1:** Basic PE or their composites influence the viruses.

Polyelectrolyte (PE) or PE-Based Composite	Virus	Ref.
Polystyrene sulfonate	Bovine papillomavirus type 1 (BPV-1), human papillomavirus type 11 (HPV-11) and type 40 (HPV-40)	[152]
Poly-L-lysine	Venezuelan equine encephalitis, Eastern equine encephalitis, human immunodeficiency virus type 1 and type 2 (HIV-1 and HIV-2), respiratory syncytial virus (RSV), Influenza A virus, Sindbis virus, Semliki Forest virus, vesicular stomatitis virus (VSV), herpes simplex virus type 1 (HSV-1), human cytomegalovirus (CMV).	[108,109]
Polyethyleneimine	Human papillomaviruses, human cytomegaloviruses, porcine reproductive, and respiratory syndrome virus (PRRSV)	[113,114]
Chitosan	Human H1N1 influenza A virus, Rift Valley Fever virus (RVFV), Herpes Simplex-1 virus (HSV-1), and Coxsackie virus	[121,122]
Hyaluronic acid	Coxsackievirus B5 (COXB5), mumps virus (MV), herpes simplex virus (HSV-1)	[119]

**Table 2:** Polymeric materials of the anti-viral activity against SARS-CoV-2. * Potential effect; lack of experimental studies.

No	Polymer	Ref.
1	Polystyrene sulfonate (PSS)	[148]
2	Poly-L-lysine (PLL)	[112]
3	Alginates	[153] *
4	Chitosan derivatives	[154,155]
5	Cyclodextrin	[156]
6	Dendrimers	[157]
7	Carrageenans	[158,159]

## Data Availability

Not available.
